# The double-edged sword of self-tracking: investigating factors of technostress in performance-oriented cycling and triathlon

**DOI:** 10.3389/fspor.2024.1465515

**Published:** 2024-11-18

**Authors:** Mirjam Werner, Andreas Bischof

**Affiliations:** Institute of Sociology, Faculty of Behavioral Science, Chemnitz Univercity of Technology, Chemnitz, Germany

**Keywords:** technostress, self-tracking, cycling, triathlon, sensors, qualitative study

## Abstract

This study is dedicated to the investigation of technostress caused by self-tracking in performance-oriented amateur sports and thus addresses a significant research gap in the understanding of stress factors operating in this specific context. Although technostress in occupational and private settings has been extensively researched, there is a lack of knowledge about the effects and specifics of technostress caused by the use of self-tracking technologies such as wearables and performance monitoring apps in sports. A total of 16 stress factors were identified, eight of which - information overload, distraction, unavailability, loss of control, lack of sense of achievement, unreliability, complexity and self-monitoring - are already known from the professional context and were transferred to the sports context. In addition, eight new stress factors specific to performance-oriented amateur sport were identified: Performance enhancement imperative, lack of context, digital visibility, feedback incorporation, measurement data fixation, comparison pressure, permanent monitoring and perception discrepancy. The study is based on a qualitative research approach with guided interviews conducted with performance-oriented amateur triathletes. The findings of this study contribute to a deeper understanding of the dynamic and contextual nature of technostress in sport and provide a basis for the development of targeted intervention strategies aimed to reducing technostress, such as adaptive training programs or personalized feedback systems. The results thus provide a valuable starting point for future research, particularly for the investigation of coping strategies in relation to the identified stress factors. In addition to identifying eight new sport-specific technostress factors, this study clearly delineates how traditional work-related technostress factors are applicable to amateur sports. This contextual adaptation helps in understanding the unique pressures faced by amateur athletes and distinguishes this study within the field.

## 1 Introduction

The rapidly advancing development of digital technologies has had a significant impact on the sports sector. One trend that is emerging not only in the professional but also in the amateur sports sector and underlines this development is the use of self-tracking devices. These wearables devices and sensors, which are worn close to the body, are primarily used to document and optimize athletic performance and are increasingly becoming an integral part of athletes’ training concepts. But do these technologies, which are often used to improve performance, also harbor potential risks (including information overload, loss of control over personal data, and increased psychological pressure related to performance), that can impair the mental and physical performance of athletes? This study addressed this question. Our contribution sheds light on the less discussed but equally critical aspects of the digital sports revolution, in particular the unintended side effects of self-tracking in cycling and triathlon. The aim is to identify stress factors that can have a negative impact on athletes’ willingness and ability to perform. This study aims to contribute to this special issue on performance enhancement strategies by highlighting factors that *reduce* performance in cycling and triathlon resulting from the use of self-tracking technologies.

Literature explicitly dedicated to the technostress-related stress factors of digital self-tracking in sport could not be identified in the course of the literature search (see 2.). This gap in the literature highlights a crucial area for exploration, as previous research such as Duttweiler and Passoth (1) and Heyen (2) has predominantly focused on the broader implications of self-tracking technologies without delving deeply into the specific stress factors induced in sports settings. The previous research on technostress caused by self-tracking mainly refers to the differentiation of user types and their characteristics as well as the effects that can result from the use of self-tracking technologies (see 1–6). With this article, we want to contribute to closing the identified research gap. As part of an exploratory, qualitative study with 13 performance-oriented triathlon cyclists (see 3.), we investigated this phenomenon and researched stress factors that can arise from self-tracking practices. This study employs a qualitative methodology to delve deeper into the subjective experiences of athletes, thus providing rich insights into the personal dimensions of technostress.

The scientific debate on the side effects of digital self-tracking technologies in sport is still relatively new and is therefore based on a limited empirical database. A comprehensive investigation of the causes, manifestations and effects of technostress through self-tracking in sport is therefore crucial to understand how technologies used to enhance athletic performance can also trigger stress and thus impair performance. To address this endeavor, we propose Lazarus and Folkman's (7) transactional stress model to analyze the complex psychological dynamics that arise from self-tracking in sport. This model provides a comprehensive framework to systematically explore the multi-layered interactions between individuals and technology and to examine in detail the key research findings on the stressors of technostress (see 2.).

The discussion of the results shows that, on the one hand, self-tracking in sport offers the opportunity to optimize training processes and monitor individual progress in detail. On the other hand, constant digital self-monitoring leads to technostress in some athletes, which can impair their well-being and performance. By gaining a deeper under-standing of the development of technostress through the use of self-tracking technologies in sport, measures can be derived in the future to predict these effects in training management and prevent them through suitable measures such as training or restricting use (see 5.).

## 2 The transactional stress model, technostress and datafication in sport

Technostress is a neologism made up of the terms technology and stress and describes a special type of stress that can be triggered in particular by the use of digital technologies (8, 9). Schmidt, Frank, and Gimpel (10) use the term “digital technologies” to refer to computer-based infrastructures, which include hardware components, software applications and their networking. Today, digital technologies are not only an integral part of economic processes, but are also increasingly conquering private areas of life, including the field of sport. This article looks at digital technologies related to performance measurement and training control, such as cycling computers with sensors, heart rate monitors and platforms for training profiles, in order to explore the stress factors of technostress that can arise from self-tracking in the sports context.

In order to approach this goal, the following chapter is first dedicated to presenting the transactional stress model according to Lazarus and Folkman (7), which is widely used in research on technostress and also serves as the theoretical basis for this study (2.1). In the next step, we examine the stress factors of technostress, which can be derived from the literature for both the professional and private context (2.2). The chapter concludes with an examination of datafication in sport and a description of the current state of research (2.3).

### 2.1 Transactional stress model and model development

**Figure 1:**
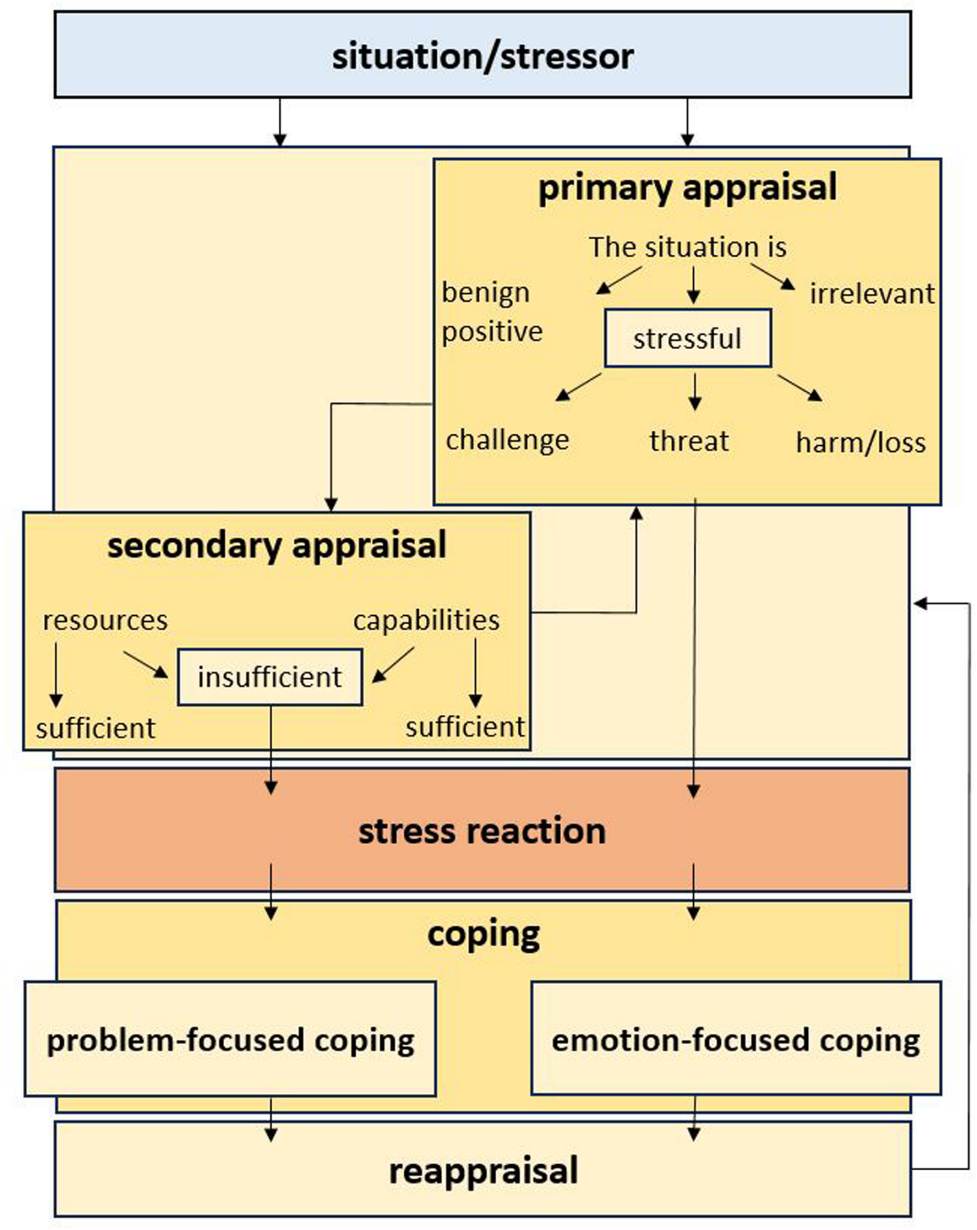
Own representation of the transactional stress model by Lazarus and folkman (7), based on zapf and semmer (11), p. 1020) and expanded according to Gimpel et al. (12, p. 19) and Rusch (13, p. 68).

The transactional stress model, developed by Richard Lazarus and Susan Folkman (7), explains the development and coping process of stress. The model shows how stress reactions are shaped by the interplay of sequential and parallel processes, whereby individual perception and subjective evaluation are decisive (see Figure 1). Cognitive evaluation processes play a central role in determining how individuals interpret and react to stressors. Thus, the same stress factor can be evaluated differently by different people, which leads to various stress reactions. This individual and subjective nature of the stress experience is a core aspect of the transactional stress model, which is discussed in detail in the following sections.

An external stimulus, which represents a potential *stressor*, flows onto an individual. The stimulus, which may be of a visual, auditory or haptic nature, is first perceived by the individual and evaluated as a potential stressor. With the first intrapersonal assessment, the *primary appraisal*, the situation is interpreted and the nature of the stressor is assessed. If the potential stressor is classified as positive/friendly (*benign-positive*) or *irrelevant*, there is no stress. However, if the stressor is classified as dangerous/*stressful*, this can mean *harm* or *loss*, a *threat* or a *challenge* and trigger a stress reaction (7, 14).

Furthermore, a *secondary appraisal* of the stressor takes place. This does not necessarily take place after the primary appraisal, but can take place at the same time or in reverse order to the situational appraisal, depending on the perception of the stressor. In the secondary appraisal, the available means for eliminating the stressor are analyzed and it is assessed whether the situation can be overcome by using personal resources or skills. If the resources are classified as sufficient, e.g., through a high level of confidence in one's own abilities or sufficient personal, social or material resources, a stress reaction can be prevented. However, if a situation is classified as dangerous and/or a lack of resources is identified, this circumstance triggers a *stress reaction* (7, 13). This can occur on the levels of physiology (e.g., through an increase in heart rate), emotion (e.g., through the feeling of fear), cognition (e.g., through the anticipation of negative consequences) and behavior (e.g., through the display of physical restlessness) (15).

As a result of the stress reaction, strategies for *coping* with stress are now discussed with the aim of potentially improving or ending the situation. Coping strategies can be *problem-* or *emotion-focused* and are to be understood as individual efforts to cope with the stressful situation - regardless of the success of the intention (16). Problem-focused coping strategies are particularly concerned with bringing about a change in the situation itself (7). In emotion-focused coping, on the other hand, the focus is on the emotional regulation of negative thoughts. The coping strategy chosen ultimately depends on the upstream assessment of the stressor, in particular the secondary appraisal. Problem-oriented and emotion-oriented coping can be used individually, but also together to potentially reduce stress (7, 17).

Following the measures taken to reduce stress, a *reappraisal* of the situation takes place. In this context, Lazarus also speaks of cognitive coping, which is evaluation-oriented. An attempt is made to change the emotional relationship to the situation, for example by reinterpreting the circumstances in a positive way (7, 17). If cognitive coping occurs as a result of failed coping strategies, an automatic feedback loop comes into effect, which is based on appraisal and coping processes that have already taken place.

The model (see Figure 1) was developed on the basis of Zapf and Semmer (11), p. 1020) and illustrates this process. It has also been extended by one level. The point of the “stress reaction”, as found in the model, was added with reference to Gimpel et al. (12, p. 19) and Rusch (13, p. 68) was added as an extension. Lazarus and Folkman's transactional model emphasizes individual appraisal of stress, but recent studies, such as Diotaiuti et al. (18), suggest that resilience and self-regulatory modes play a crucial role in moderating stress responses in endurance athletes. This further supports the need to consider psychological resources when analyzing technostress in performance-oriented sports.

The transactional stress model according to Lazarus and Folkman (7) is often used in technostress research to analyze the causes and contexts of technostress (8, 9, 19–22). Following on from this, the present study focuses on the stress factors that can arise from self-tracking in sport and thus cause technostress. According to the model, these are not the stressor itself, but can be derived from the primary appraisal of the individual: In other words, what already appears potentially threatening to the users of digital technologies. The following chapter takes a detailed look at the stress factors already identified in the literature in order to gain a deeper understanding of their role and influence on technostress.

### 2.2 Stress factors of technostress

The aim of the study is to investigate the stress factors of technostress that can arise from self-tracking in a sports context. In order to approach this goal, the following chapter is first dedicated to presenting the stress factors of technostress that have been researched to date.

**Figure 2:**
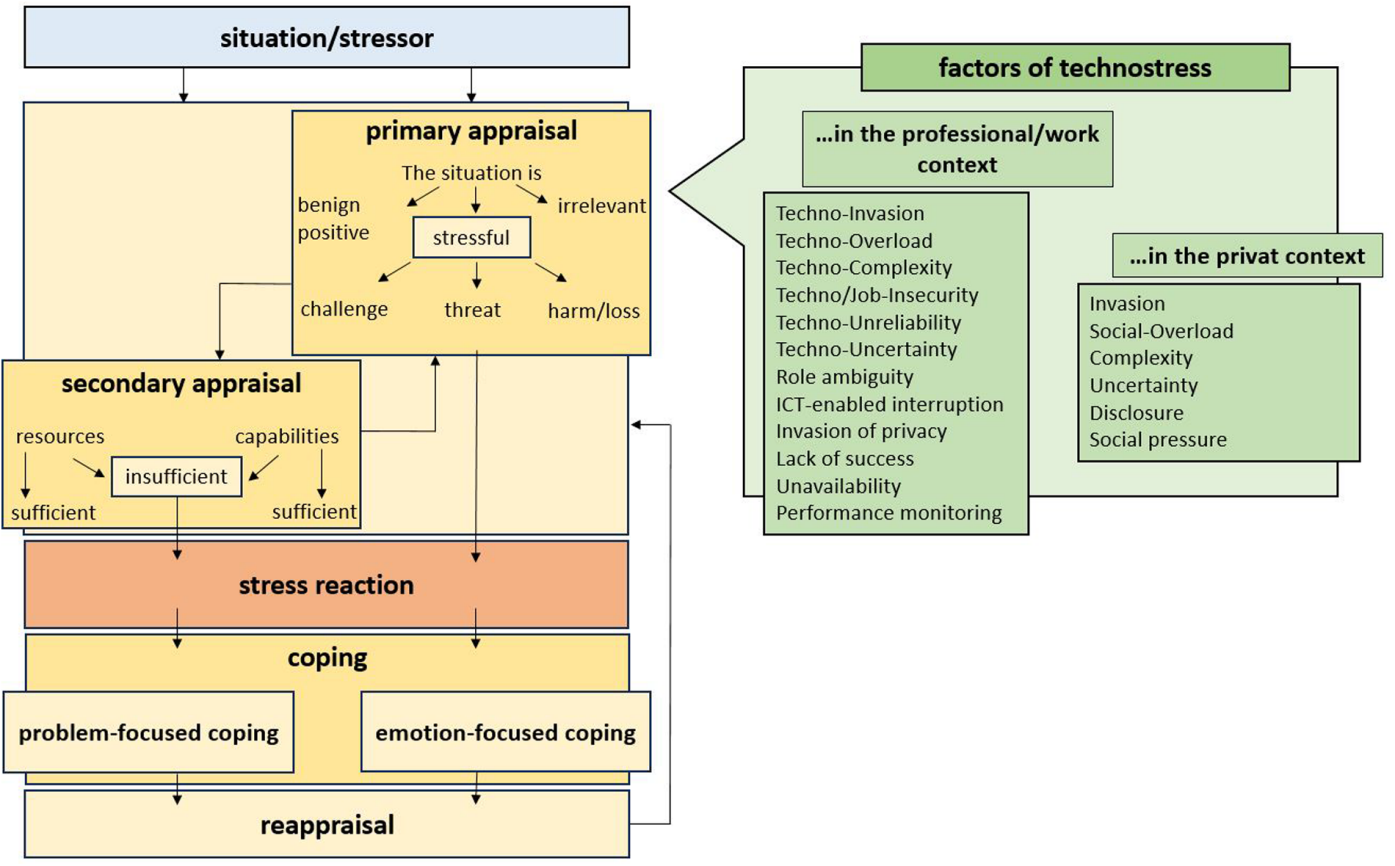
Own representation, based on the stress factors identified in the literature (green box).

In technostress research, two strands of research can be considered: the investigation of technostress in a professional context and the analysis of technostress triggered by the private use of ICT. Figure 2 shows such a classification. The findings of existing research on professional and private technostress were classified as factors of *primary appraisal*. These are shown in the green box. On the left-hand side are the factors of occupational technostress that have already been researched. On the right-hand side are the stress factors of private technostress that have been identified in the literature (see Figure 2). Recent studies have emphasized the importance of ensuring measurement invariance and psychometric reliability when using self-assessment tools in psychological research (23).

As a result of technological progress and the associated implementation of information and communication technologies (ICT) in the workplace from the mid-1980s, technostress research initially concentrated on the investigation of technology-induced stress in connection with gainful employment (24, 25). In contrast, research into factors that trigger technostress in a private context has only recently become the focus of scientific discourse. In particular, the consequences of using smartphones and social media have been studied in many cases.

The combination of technostress research in relation to organizational and personal ICT use has produced an extensive list of technostress contributors. This section presents the stress factors identified in the course of the literature review that occur in both professional and private contexts (see Table 1). The stress factors that trigger technostress in a private context have been little researched in comparison to those in a professional environment. So far, only one specific stress factor, social pressure, has been identified. Maier (30) has been identified as exclusively relevant to the private sphere. All other factors discussed in the private sphere were originally investigated in a professional context and later transferred to the private sphere. Our research aims to expand this list. In particular, no specific factors could be identified in the literature review for the stress-inducing factors arising from the use of wearables in sport.

**Table 1:** Own presentation, based on the stress factors of technostress identified in the literature.

Factor	Description	Source
*Invasion*	Includes all factors in which ICT leads to or facilitates conflicts between the digital and offline worlds. This impairs the desired demarcation of an area of life without the influence of ICT. Invasion can create the feeling of having to be constantly available, having a high level of responsiveness or being permanently confronted with information and communication technologies.	Techno-Invasion (9) Work-Home-Conflict (8) Invasion (26)
*Overload*	Describes the stressful situations that can arise due to the large number of tasks and external requirements in the context of using information and communication technologies (ICT) and thus overtax the user.	Techno-Overload (9) Work overload (8) Social overload (26)
*Complexity*	Arises when the complexity of the usability of ICT is considered too high to be mastered with one's own skills and resources. As a result, users are forced to compensate for the mismatch between skills or knowledge and the given requirements.	Techno-Complexity (9) Complexity (26)
*Insecurity*	Describes the fear of losing one's job due to advancing digitalization and automation. These ICT factors threaten users’ future prospects by fueling fears of being replaced by more tech-savvy colleagues or new technologies, which severely affects job security.	Techno-Insecurity (9) Job insecurity (8)
*Uncertainty*	Describes the constant challenge of dealing with the fast pace and frequent changes in technology that affect users of ICT. This uncertainty is compounded by the need to continuously learn and upskill in order to keep pace with the constant changes and updates in technology.	Techno-Uncertainty (9) Uncertainty (26)
*Unreliability*	Describes the burden placed on users by unexpected errors in information and communication technologies. These errors can be, for example, system crashes, unstable applications or long loading times, which promote the perception that ICT is unreliable. Such unpredictable malfunctions affect the user experience and the handling of ICT	Techno-Unreliability (8, 27)
*Interruption*	In the work task caused by the transmission of spontaneous messages (such as e-mails, phone calls, etc.) and information can trigger stress.	ICT-enabled interrution (28)
*Disclosure*	Describes the fear that one's own privacy will be violated by the use of information and communication technologies at work or in the private sphere, for example due to unclear data protection settings or a lack of transparency in data processing.	Invasion of privacy (8) Disclosure (26) Privacy concerns (29)
*Role ambiguity*	Describes an unintentional postponement of the actual work task when digital technologies fail at the workplace and the malfunction has to be rectified by the employee's own efforts.	Role ambiguity (8)
*Performance monitoring*	Describes the fear of being constantly monitored and evaluated by information and communication technologies in the workplace.	Performance monitoring (12)
*Unavailability*	Describes the feeling of not being able or allowed to use digital technologies that could, for example, make it easier to solve problems or delays in the work process	Unavailability (12)
*Lack of success*	Describes a subjective feeling of achieving little or no progress in work through the use of digital technologies, as digital progress can sometimes be less visible.	Lack of success (12)
*Social pressure*	Describes situations in which individuals are pressured by their social environment to use information and communication technologies in a certain way or to adopt certain behaviors.	Social influence (26)

In this research project, the focus is on self-tracking technologies that are used to measure personal body and performance states in sport. It should be noted that sporting activities, although often assigned to the private sphere, have characteristics of work in performance-oriented amateur athletes. These include not only physical exertion and systematic planning, but also the continuous monitoring and analysis of performance data, which can be reminiscent of professional activities. In addition, participating in competitions or adhering to strict training schedules often requires a disciplined approach that makes similar demands to professional projects. These overlaps show that a clear separation between professional and private contexts in relation to technostress is not always possible and requires a differentiated view of the phenomenon, especially in performance-oriented recreational sport. The state of research on datafication in sport is therefore presented in more detail below.

### 2.3 Datafication in the sports context

With the advancing technical development and the introduction of digital technologies into the field of sport, rationalization, quantification and optimization practices are now taking place (31) that were previously analog and manual are now increasingly automated and digital (32–34). In the sports and fitness sector, for example, digital self-measurement technologies can be found in the form of smart bracelets, watches, (swimming) goggles, jewelry, but also sensors that can be attached directly to the skin (e.g., by gluing) or, in some cases, implanted (35, 36). The sensors measure a wide range of parameters and can thus provide information about the individual performance values or conditions of the body. The measurable variables include, for example, heart rate, calorie consumption, sleep and recovery quality as well as the number of steps taken or distance covered (37, 38). However, the reasons why people choose to use self-tracking vary - as do the consequences of using it.

#### 2.3.1 Motives for the use of self-tracking technologies

The motives for using self-tracking technologies reported in the literature can be divided into three overarching categories based on Suh (6): hedonic, utilitarian and eudaemonistic. This categorization serves to systematically record the diverse motives for use and to put them in relation to each other.

1. **Hedonic motives** relate to the use of self-tracking technologies to increase personal pleasure and well-being. The focus here is on the immediate pleasure and enjoyment of the activity, without actively pursuing a goal (6):
  • **Fun or entertainment**: users seek pleasure in the activity itself (1).
  • **Health improvement** can also have hedonic aspects if the goal is to increase one's own well-being (3, 4).
2. **Utilitarian incentives** focus on the practical usefulness and functionality of self-tracking technologies. This category includes motives based on clear objectives and the pursuit of efficiency (6):
  • **Performance enhancement:** Targeted use to improve one's own sporting or health performance (1).
  • **Classification and legitimization of sporting performance:** Using technology to objectively assess and recognize athletic performance (39–41).
3. **Eudaemonistic motivation involves** the use of self-tracking technologies to promote personal development, identity work and self-realization. It is about a deeper sense of purpose and the pursuit of individual excellence (6):
  • **Self-expertization**: Gathering detailed information about one's own body in order to gain comprehensive self-knowledge (42).
  • **Self-empowerment**: Using technology to empower autonomy and control over one's health and performance (43).
  • **Identity formation and work**: Self-tracking as a tool to support one's own development history and role-finding in sport (33, 44–47).
  • **Health improvement** can also fall under the eudaemonistic category if it is aimed at the pursuit of self-development and personal excellence (3, 4).

This classification shows that the motives for using self-tracking technologies are multifactorial and interlinked. While some motives can be clearly assigned to one category, others overlap in nature and can be located in more than one category, as the example of health improvement shows. Here, the literature distinguishes, for example, between the motivation to feel the improvement in performance physically (hedonic motivation) or to want to achieve it as part of identity work (eudaemonistic motivation). The categorization into hedonic, utilitarian and eudaemonistic motives therefore provides a useful framework for understanding and systematizing the complex motivations of users.

#### 2.3.2 Effects of the use of self-tracking technologies

In addition to the motives for using self-tracking services, the psychological, psychosocial and physical *effects* that can arise from the practices of digital self-tracking have also been scientifically investigated and frequently addressed. The following effects of self-tracking use in the context of sport have been identified:

• Reduced enjoyment of movement and the environment (33, 48, 49)
• Loss of intuitive body awareness of the signs of health and illness (49, 50) or devaluation of one's own subjectivity and inwardness (21, 44, 51)
• Perception of being dominated or controlled by numbers (8, 52)
• Fears and doubts (53)
• Emotional stress as a result of a feeling of pressure from continuous activity (50, 54)
• Frustration caused, for example, by a different experience between the data obtained and personal perception (55, 56)
• Development of a dependency on self-tracking technologies (55, 57–59)
• Objectification of self-perception (5, 60–62)
• Emotionalization, triggered by the interpretation of individual performance data. Emotions can range from joy and pride to anger, shame and guilt (39, 63)
• Mobilization through special application offers, such as memory functions and performance comparisons as well as an increase in social control over (sports) behavior (39, 64)
• Sports addiction, which expresses itself in an excessive urge to exercise (65)
• Motivational problems that manifest themselves in a reduction in physical activity (36, 57)
• Transformation of sports practices through the inclusion of self-tracking technologies, towards digitally measurable activities (5, 39, 58)
• Technization and virtualization of interpersonal relationships, as people are reconnected through the integration of digital self-tracking technologies, e.g., by establishing digital sports-related competitive relationships through the publication of personal performance data on digital (sports) platforms (1, 47, 66, 67).

The results presented show that research into the motives and practices of use and the effects that can result from the use of self-tracking technologies has been investigated many times. In addition, the results make it clear that there is a consensus regarding the datafication of everyday life that digital data from everyday activities and social life interact reactively and thus influence each other (68). Concrete findings for stress factors that cause technostress and result from the use of wearables in sports for athletes could not be identified in the course of the literature research.

### 2.4 Exploring technostress in (recreational) sport with the transactional stress model

Building on the existing findings, this study aims to investigate the practice of self-tracking in the sports context and, in particular, to identify the stress factors of technostress generated by self-tracking. The transactional stress model according to Lazarus and Folkman (7) serves as a framework for differentiating and analyzing individual stress reactions and coping strategies from the stress factors to be identified. Although the model enables a differentiated view, its applicability in the context of digital self-measurement must be critically evaluated, as it may not fully reflect the specific dynamics of technological interactions. The suitability of the model for the issue at hand is therefore discussed below.

The transactional stress model is beneficial for an empirical study in that it emphasizes the subjective perception of stressors. This is relevant because the perception and evaluation of self-tracking technologies strongly depend on individual factors such as personal goals, experiences and attitudes towards technology. In addition, the model enables a differentiated view of coping strategies. In the field of sport, where self-tracking tools can be both beneficial and detrimental, it provides a framework for under-standing how athletes deal with the stress induced by technology and what strategies they develop to manage, for example, performance pressure and surveillance anxiety. The decision-making levels proposed in the model are of particular importance here, as they should guide the structuring and classification of the data collected. The clear subdivision of the transaction process into small steps can help to differentiate the athletes’ technostress experiences more sharply and thus map them in more detail.

A major criticism of the application of the transactional stress model to technostress through self-tracking in sport is that it may not fully capture the specific characteristics of technological interactions. Self-tracking in sport involves not only direct interaction with technology, but also continuous self-observation and self-assessment based on data and its visibility, which can introduce new dimensions of stress that are not explicitly considered in the model. The rapid advancement of self-tracking technologies and the constant presence of digital data can become a source of stress that goes beyond the traditional view of stress as a reaction to specific, identifiable stressors.

Furthermore, the transactional stress model may be too individual-centered and does not sufficiently take into account the social and cultural contexts in which technostress occurs. In the field of sport, the use of self-tracking technologies can be influenced by cultural norms regarding performance and health as well as by social comparison processes, which modify the individual experience of stress. Another limitation is the model's assumption that stress experiences are always clearly defined and conscious. In the context of self-tracking, however, technostress can arise through subtle and unconscious processes, such as the creeping normalization of continuous performance monitoring, which the model may not fully address. Also, factors in the technological context, such as the speed at which data is collected by wearable devices, are not considered in the model.

Despite the above criticisms regarding the application of Lazarus and Folkman's (7) transactional stress model, the model offers valuable insights into the processes of stress perception and coping and can provide a useful theoretical framework for the study of technostress through self-tracking in sport. However, the specific characteristics of the technological interaction and the context in which these technologies are used need to be carefully considered. For this reason, the Transactional Stress Model will not be the focus of a deductive approach in which the theory is tested for its validity in the area of technostress in (recreational) sport. Rather, it will be used as an exploratory tool, the value of which lies in gaining a structured derivation of the collected data and a deeper understanding of technostress-inducing stress factors through self-tracking, especially in ambitious triathletes and cyclists.

## 3 Study & method

Athletes who practise triathlon as their main sport were selected as the survey group for this study. Triathletes primarily practice three sports disciplines, swimming, cycling and running, within which digital self-tracking technologies can be used for personal analysis. With regard to the study results by Gimpel et al. (12) and Ragu-Nathan et al. (9) which show that an increasing degree of digitalization in the workplace is associated with an increase in perceived technostress, it was assumed that triathletes who practice three digitally measurable sports are potentially exposed to more stress factors of technostress than athletes who only practice one digitally measurable sport, for example. Based on the formulated research interest, ambitious triathlon is therefore a suitable field of research in which it can be assumed that the phenomenon of technostress through self-tracking comes to light. The sample was drawn up with reference to the defined field.

### 3.1 Sample & recruiting

Only people who practice triathlon at an amateur level were included in the study. *Amateur athletes* in the context of this study, refers to individuals engaged in competitive sports outside of a professional framework, with a primary motivation of personal development rather than financial gain (69, 70). In contrast to *professional athletes*, who earn their own living through the active and organized practice of a particular sport (71, 72) amateurs practice their sport for its own sake, without gaining any material or monetary benefits (69, 73). In the preliminary consideration, it was assumed that professional athletes use self-tracking technologies as work equipment and therefore have a different relationship to those technologies than is the case with amateur athletes. Amateurs voluntarily use the services of digital self-tracking technologies without this being a (professional) necessity.

Furthermore, only amateur athletes who participate in triathlon at a competitive level were surveyed. *Competitive sport* is defined as sporting activities that can be assessed on the basis of certain quality criteria (e.g., time, distance, weight, height). The defined goal of competitive sport is to improve personal performance, which is achieved through performance-enhancing activities (hard training, abstaining from stimulants such as alcohol, etc.) (72, 74). In contrast to other forms of sport, such as *recreational and popular sports*, which tend to focus on playful sporting activities (72, 75) the competitive principle applies in competitive sport, particularly in the context of competitions. *High-performance and elite sport* is defined as the higher level of competitive sport. This is recruited from the elite athletes of competitive sport. One of the characteristics of this group of people is that they focus their lives and activities on improving their performance in pursuit of world records. The aim is to be as competitive as possible for a certain period of time and to be able to dominate the international field (76, 77).

In accordance with the formulated research interest, the aim of the study is to determine the stress factors of technostress in individuals who engage in self-tracking particularly frequently (actively) and extensively (passively) in training and competition. Due to the performance-oriented focus of this group of athletes, the personal focus is on their own performance and its development (72, 74). The use of self-tracking technologies, which carry the promise of continuous self-measurement, should therefore be used particularly frequently by the group of competitive athletes in terms of self-monitoring.

Furthermore, access to the research field was a deciding factor. Experience has shown that performance-oriented triathletes are often organized in clubs in order to benefit from the available training opportunities and association structures. This is only partially the case for amateur and recreational athletes who practice triathlon at a playful, non-performance-oriented level. Access to this group would therefore have been problematic. The same applies to high-performance and elite athletes. Although they are usually affiliated with a club, they often act separately. In addition, this group is heavily interspersed with professional athletes, which is why it was decided not to look at this group of people.

The aim of the study was to investigate the phenomenon in question on various dimensions and to explore the different facets of technostress through self-tracking. In order to achieve this, the sample was designed to be as contrastive as possible. This means that the cases examined should differ as much as possible within the defined field in order to be able to take into account and depict the broadest possible spectrum of technostress-related experiences. The contrastively selected categories relate to age, gender, home club and training volume.

A total of 13 qualitative interviews were conducted. When selecting the cases, the percentage distribution of the current survey of the German Triathlon Union was used as a guide wherever possible. This determined 68.5% male members and 31.5% female members for the year 2023 (78). This distribution was approximated by including five women (−38.5%) and eight men (−61.5%). The age range of the respondents is between 24 and 64 years. The average age is 37.8 years. When selecting the cases, care was also taken to ensure that the interviewees came from as many different clubs as possible and were therefore in different training environments. For this purpose, triathletes from seven clubs were recruited, four of which are located in Saxony, one in Bavaria, one in Baden-Württemberg and one in North Rhine-Westphalia.

The competition distance at which the interviewees are competing (or training) and the associated training volume are also different. Two triathletes compete in short-distance races, two in middle-distance races and nine in long-distance races. During the survey period, the athletes’ lowest training volumes were between six and eight hours per week, while the highest volumes were between twelve and 16 h. On average, the triathletes surveyed stated a weekly training volume of 9.5–12.8 h. It should be noted that the interviews were conducted from February 2023 to May 2023. Typically, triathletes actively work towards one, or at most two, seasonal highlights and focus their personal training on these. In terms of periodization, the training volumes vary, build up over certain periods of time, shift their focus and are reduced again at a given point (79–81). Due to the time of year in which the interviews were held, eleven of the 13 interviewees were in training, which is why relatively high training volumes were reported in some cases, especially by the long-distance athletes (up to 16 h per week). If the same athletes were to be interviewed again six months later, they would probably state significantly lower training volumes (see Table 2).

**Table 2:** Representation of participants by gender, age, training hours per week, association and race distance.

TN	Gender (f/m/d)	Tracking system	Age	Ø training hours/week	Association	Race distance
*T1*	f	Garmin, Wahoo	24	6–10	A	Sprint distance
*T3*	m	Polar	42	6–8	A	Sprint distance
*T4*	m	Garmin	30	10–13	B	Full distance
*T6*	m	Garmin	34	12–16	C	Full distance
*T7*	m	Garmin, Wahoo	42	12–15	A	Full distance
*T9*	f	Garmin	33	12–16	B	Full distance
*T11*	f	Garmin	30	10–12	D	Full distance
*T13*	m	Garmin	45	8–12	F	Full distance
*T14*	m	Garmin	49	8–12	G	Full distance
*T15*	m	Garmin	64	10–14	H	Full distance
*T19*	f	Garmin	32	12–14	B	Full distance
*T21*	f	Garmin, Wahoo	28	8–12	D	Middle distance
*T23*	m	Wahoo	39	10–12	A	Middle distance

Access to the research field was gained in various ways. First and foremost, access was made possible through direct contact with the home association. The literature draws particular attention to the potential difficulties that can arise when close persons are involved in the research (82, 83). It was therefore important to approach athletes outside the direct training group with whom contact was only sporadic. Another access route was via the chairmen of other triathlon clubs in Saxony, who passed on the request in their training groups and established the relevant contacts. There was also the opportunity to accompany a triathlon training camp on Mallorca, which proved to be extremely valuable for data collection. This training camp was aimed at performance-oriented long-distance triathletes with the season highlight of Challenge Roth at the end of June 2023. The participants provided written informed consent to participate in this study. Future studies could enhance the generalizability of these findings by incorporating a larger and more diverse sample, thus providing a more comprehensive view of technostress across different sports disciplines.

### 3.2 Methods & data

A total of over 17 h of audio material from qualitative interviews with 13 athletes was secured. The average duration of the interviews was 80 min, ranging from 67 to 99 min. All recorded interviews were converted into writing using speech recognition software. Erroneous passages, such as those caused by slurred speech or dialects, were then corrected by hand. The transcripts were software-supported, using qualitative content analysis according to Mayring (84) analyzed. The data was analyzed using MAXQDA, a qualitative content analysis software, and the stress model by Lazarus and Folkman was applied to categorize responses into primary and secondary appraisals of technostress. The reliability and validity of the self-report questionnaires used in this study are supported by previous research on psychometric tools in similar contexts (23).

An integrated approach was deliberately chosen for this study, which included both deductive and inductive methods of data analysis. This decision was based on the realization that such an approach enables a more comprehensive understanding of the research subject to be gained. By combining deductive and inductive approaches, both theoretical concepts and empirical data could be used to support the exploratory nature of the analysis.

With the help of deductive methods, the structures of the transactional stress model were integrated as existing theoretical concepts. This integration proved to be extremely helpful, particularly for differentiating the stress factors from other characteristics such as stress symptoms or coping strategies. On the other hand, the stress factors of technostress already identified in the professional and private context were used as a starting point for the study in order to test their transferability. This inclusion made it possible to develop the theoretical framework that structured the analysis. An inductive approach was also integrated. This enabled an in-depth examination of the empirical data and the pursuit of new findings and correlations.

To this end, relevant passages were extracted from the transcripts of the interviews that showed a connection with the negative assessment of the (non-)use of self-tracking technologies in triathlon. These categories were then successively generalized and abstracted before being structured into super- and subcategories. A total of 1,575 codes were assigned in the data analysis.

In summary, it can be stated that a qualitative research design was used for this study, which was exploratory in nature. The data was collected with the help of guided, problem-centered interviews, which allowed for an open discussion. However, there are various limitations, including the distortion of response behavior due to social desirability and interviewer effects. In addition, the use of interview guidelines makes the results less comparable. The sample is made up of performance-oriented amateur triathletes who actively and passively engage in self-tracking. The categories of age, gender, home club and training volume or competition distance were selected for contrast. Access to the field was via the club structures of their own home club, the referral of other triathletes by under-suited club presidents and through participation in a training camp for performance-oriented long-distance athletes.

## 4 Results

**Figure 3:**
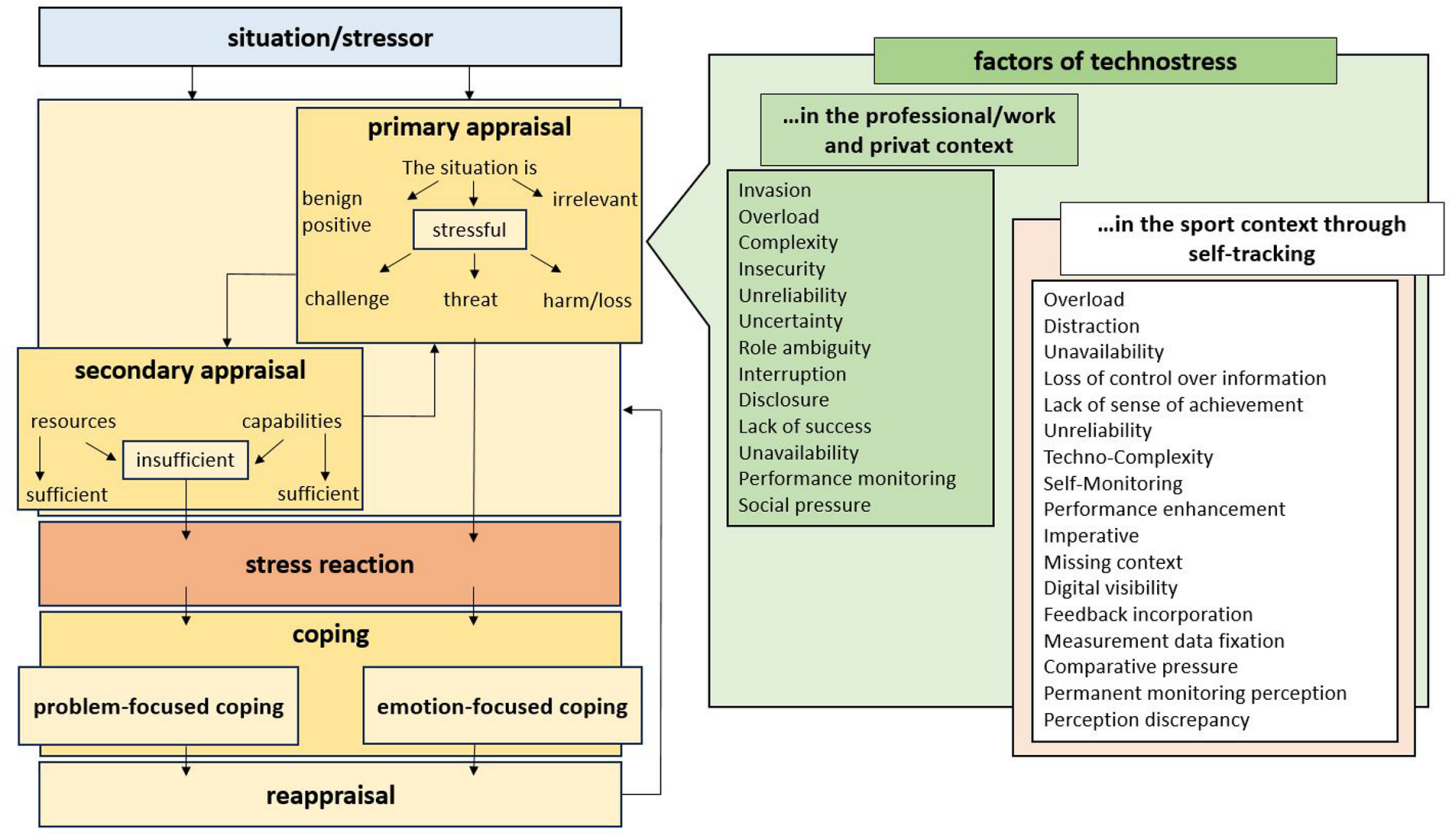
Own representation, based on the stress factors identified in the literature (green box) and expanded to the confirmed and newly explored factors of technostress through self-tracking (red box).

The evaluation of the interviews confirmed eight of the twelve stress factors of technostress that had already been identified in the professional and private context. In addition, eight further stress factors were identified. This chapter presents the confirmed stress factors and the new findings. Figure 3 graphically illustrates the new findings (red box), which contribute to the expansion of the existing body of knowledge (green box).

### 4.1 Confirmation of known stress factors

In the interviews, eight of the stress factors of technostress known from the literature could be applied to self-tracking in performance-oriented recreational sports and contextualized accordingly. Although these stress factors were originally defined for a different context, as described in Section 2.2, they could be transferred to the present circumstances without fundamental reinterpretation. In the following, these factors are briefly presented and summarized with the key findings.

#### 4.1.1 Overload (codes assigned: 19)

Wearables continuously collect data and can break down sports sessions into small amounts of data. If this data is retrieved or displayed in real-time during the session, athletes may feel overwhelmed by the constant influx of information, disrupting their concentration and performance. The information overload can be triggered by sport-related and non-sport-related data. Sports-related data refers to information that is related to the sports session performed. This can trigger stress immediately (during the sports session) or with a time delay (retrospectively, after the sports session). “I even thought about it yesterday, maybe I'll do it now in [Challenge] Roth on the first lap, tape off my speedometer” (T13, lines 340–342), reports one athlete about his attempts to reduce the flood of information. Non-sport-related data refers to information that can be forwarded from the smartphone to the wearable, such as emails, call notifications or text messages from social networks or messaging services. “The [watch] is always in front because I always get distracted because messages come in and I look at it all the time”. (T19, lines 30–32).

#### 4.1.2 Distraction (assigned codes: 12)

Athletes run the risk of being distracted during a sports session by the transmission of spontaneous messages (such as emails, call notifications or other text messages) or the checking of current performance data in their concentration on the movement sequences to be performed. This information can be perceived as a distraction. The distraction can affect the quality of training, trigger stress and become a safety risk in threatening scenarios, e.g., through distractions in road traffic. One athlete reports: “And if you want to constantly orientate yourself according to your wattage and you somehow need this feedback, then you only ever look at the speedometer, only ever look at the speedometer, or at the clock. Then you're distracted from it. And that's why I think it's dangerous”. (T3, lines 225–229) or “So when you see it like that, when you're swimming - people come - they're soon banging against the side of the pool because they have to press [the watch], right?” (T15, lines 377–379).

#### 4.1.3 Unavailability (codes assigned: 61)

The stress factor of unavailability is felt when athletes either do not have access to their tracking gadgets (e.g., because they have forgotten them) or do not have access to certain digital applications (e.g., due to technology failure) that are important for recording or performing their sports session. This feeling is accompanied by frustration and a sudden loss of motivation. In addition, the absence of tracking gadgets can limit the athletes’ ability to act. Some athletes are dependent on automatically loaded training plans, which are not accessible if the gadgets are unavailable. This circumstance can lead to uncertainty and inability to act. When asked what has changed for her since she started recording her sports sessions, one sportswoman replied: “In any case, if you're doing sport and you start running and realize, “Oh, shit, I've only got 5% [battery] left” or something, then you'd rather turn around and put the watch on than say you're going to keep running. Because then you have to upload something. That would be a waste - that's really stupid! It would be a waste if you went on running for another three quarters of an hour. Yes, then you'd better plug it in. Or if the bike computer runs out, then I'd rather ring the doorbell at some household, “Here, can you maybe charge my thing somehow, because- Otherwise the 50 kilometers are gone”, in the middle of nowhere. That's snot, but that's so right- That would be an option at the moment”. (T1, lines 453–463).

#### 4.1.4 Loss of control over information (codes assigned: 31)

Athletes who use self-tracking gadgets consciously and unconsciously generate digital data that not only provides insights into their physical conditions and performance, but also captures information such as location data, behavioral patterns and personal identifiers. This realization can raise fears of possible misuse of the data and lead to a feeling of loss of control over one's own information. This loss of control can be experienced on three levels: firstly, through unauthorized access by third parties, for example through data theft in hacking attacks. An athlete who holds a managerial position in a software company reports: “I have employees who can hack any Facebook account, right? That's not the problem. So it's all very open, isn't it? If we can do that - and then there are companies that do nothing else”. (T15, lines 1,051–1,053). Secondly, the perceived loss of control can be suggested by the provider of the operating software itself. “That's dangerous. You're not actually allowed to do that. I think it's very dangerous that the data is sold to insurance companies and that will be a huge problem”. (T13, lines 1,101–1,103). And thirdly, the release of personal data by the athletes themselves is a factor in the loss of control they experience. The associated uncertainty and ignorance regarding the further use of their data can cause stress among athletes. For example, several athletes reported being afraid of break-ins due to the publication of GPS and location data. “What I had already done once was to hide the start and finish because of data protection. But that was more due to the fact that nobody knows where the time trial bike is”. (T7, lines 1,120–1,122).

#### 4.1.5 Lack of sense of achievement (codes assigned: 5)

Wearing wearables can make a significant contribution to the stress factor of the lack of a sense of achievement in self-tracking. Through continuous reminders of set goals and constant monitoring of performance, it can increase the pressure to achieve these goals. Continuous data collection and real-time feedback can inform the athlete immediately if personal performance falls short of expectations. It also enables comparison with others, which can trigger and increase pressure to keep up or improve. The constant presence of the wearable and the associated expectations can lead to a heightened perception of a lack of success and increase the athlete's stress level. One athlete who has ambitions to lose weight, for example, says: “And so this tracking, as long as it works, it's like an incentive. But if it doesn't work, then it's also really frustrating, because you really - you measure yourself and sometimes it doesn't work at all. You don't do anything else. You don't eat carbohydrates, you diet, but sometimes your body just doesn't lose weight”. (T14, lines 622–627).

#### 4.1.6 Unreliability (codes assigned: 21)

If wearables have faulty functions or are even unstable in their use (e.g., in the form of technical failure), the stress factor of unreliability comes into play. Athletes experience their tracking gadget as unreliable and not useful for their project. The feeling of unreliability is accompanied by a feeling of helplessness and a loss of trust in the technology and can trigger technostress as a result. One athlete reported: “[..] or there are also catastrophic moments when the battery of the chest strap or something suddenly runs out during a run and you suddenly no longer have a heart rate”. (T3, lines1333–1,335).

#### 4.1.7 Techno-Complexity (assigned codes: 16)

The feeling of techno-complexity is felt when the usability of the self-tracking gadgets is classified as complex and exceeds one's own abilities. On the one hand, this relates to the usability of the devices themselves. On the other hand, the concept of complexity refers to the understanding of the data and calculations that the tracking devices provide for self-analysis. One athlete reported: “Wattmeter - I changed the battery on my bike. You have to use this little thing - now it spits out stupid values, doesn't bother me that much, but shows strange values in the measurement - so in the app. Doesn't put me under pressure. But it still bothers me because I don't have the expertise to change it myself. My friend has to do it. I can - I don't have the expertise to deal with it all the time either - he does my update too. I don't feel like dealing with it. I'm a real tech Mareike who has all that, but would need a trainee to set it up properly”. (T19, lines 865–873) Another athlete said: “It's also difficult - well, I tried to read up on heart rate variability, how it works or what it is. But it's such a complex subject. I honestly didn't understand it”. (T7, lines 352–355).

#### 4.1.8 Self-Monitoring (assigned codes: 34)

The stress factor of self-monitoring describes the pressure that people feel to constantly monitor themselves, both in terms of their sporting performance and their physical condition. This intense feeling of monitoring can lead to a reduced body awareness. Affected athletes express the need to be continuously informed about their performance and physical conditions, accompanied by the fear of not noticing changes in their values in time. In addition, it can cause stress if the tracking device is temporarily unavailable (e.g., during charging) and self-monitoring has to be interrupted for a short time. As an example of this, one athlete reports on the period when his watch is on the charger: “But I also think it's nice not to have anything on my hand. But it's like - you have it in the back of your mind that it's not collecting any data about you now”. (T4, lines 548–550) Another athlete also talks about the unavailability of familiar functions as follows: “What also really stresses me out is when I somehow don't have internet or there's some kind of disruption on Strava or Garmin and things aren't uploaded directly when I press the Bluetooth connection button and then it takes ages to upload. So I had a situation like that on vacation, I spent two hours trying to get it up there somehow. And it's actually completely idiotic, because sacrificing two hours of vacation to upload some stupid thing”. (T21, lines 880–886).

### 4.2 Results for the expansion of technostress research

In addition, eight stress factors were identified, which expand the existing literature base. These are as follows:

#### 4.2.1 Performance enhancement imperative (assigned codes: 17)

The stress factor of the performance enhancement imperative describes the pressure or expectation to constantly improve personal performance or achieve certain goals that are monitored through the use of tracking devices. This often leads to a drive for constant improvement and can result in stress, dissatisfaction and excessive training to achieve the set goals. If performance levels fall, this is perceived as a burden. For example, one athlete answers the question of how it feels when the watch assesses their personal training status as a “loss of form”: “Oh, that's really bad. Yes, that's not good. That's really bad”. (T6, line 523) The feeling of the performance enhancement imperative can be triggered simply by putting on the wearable. It also shows that athletes particularly prefer to record and save units that serve the goal of digitally mapped performance improvement. When asked when it feels good to record sports sessions, one athlete replied: “I can only really tell afterwards. If the session went well, then it was great that I recorded it”. (T21, lines 303–305).

#### 4.2.2 Missing context (assigned codes: 15)

The stress factor of the lack of integration of personal contexts describes the tension that tracking devices only record objective data and take no account of personal, subjective circumstances (such as illness, menstrual cycles, lack of time), which in real life are often seen as a legitimizing factor for drops in performance. This can lead to frustration among athletes if their subjective experiences and circumstances are not taken into account when measuring performance. One athlete talks about her digitally determined training readiness through the sports watch as follows: “Even if the [watch] says “Great today!”, you can feel like shit. You can think about things in your head. So you can't trick your-self. It still doesn't work. If you have your period, if you have a stomach ache, if you have a blister on your foot, the watch doesn't know that”. (T19, lines 589–592).

#### 4.2.3 Digital visibility (codes assigned: 64)

The stress factor of digital visibility describes the pressure caused by the disclosure of personal data and activities on platforms such as Strava, where profiles and usage data are automatically transformed into diagrams and analyses. This data can be viewed by others and provides a deeper insight into the body's own processes than would be possible in real life. Through cross-linking with other platforms, such as sharing Strava up-dates on Instagram, this can lead to athletes engaging intensively with their digital self-image and feeling constant pressure to shape and maintain this image. This can lead to stress, as digital visibility is often performance-related and the pursuit of positive proof of performance and progress requires constant effort. The following comments from an athlete underline this stress factor: “Although it also stresses me out and annoys me that the [indoor trainer] always shows five watts less on average than my power meter crank, yes, I mean, that's still all within limits, it's all within the tolerance range. But it's not so nice. Yes, and since I upload it all to Strava - yes, of course it's all there and people can see it all”. (T9, LINES 77–81).

#### 4.2.4 Feedback incorporation (codes assigned: 89)

The loading factor of feedback incorporation describes the tendency of athletes to accept and internalize the feedback and performance assessments of their tracking devices as authoritative assessments of their own performance. This incorporation of feedback is often seen as a direct indicator of personal performance, whereby the measured data becomes the basis of one's own performance perception. One athlete, for example, describes how she overwrites her previously perceived strong units as a result of an increased heart rate display and adapts her interpretation to the digital evaluation: “Even if it just feels good and so, from running, but the pulse is not right. That's- There are days like that from time to time and that's- You kind of ruin your run again, or your bike ride”. (T21, lines 868–872). This perception means that tracked events can be perceived as more valuable than non-tracked activities. Negative evaluations can also influence self-perception and identity as an athlete and contribute to athletes looking for explanations to distance themselves from the evaluations they perceive as negative. Positive feedback, on the other hand, can lead to a strengthening of identity and motivation. Ultimately, feedback incorporation is closely linked to athletes’ self-perception, identity formation and behavioral adaptations and can reinforce the performance enhancement imperative, which can subsequently lead to technostress.

#### 4.2.5 Measurement data fixation (assigned codes: 113)

The load factor of measurement data fixation refers to the phenomenon in which athletes allow their movement decisions and intensities during training or competition to be strongly influenced by the technical data of their tracking device. This can lead them to make their sports units more or less intense in order to achieve their digital goals, such as a round kilometer count or achieving best times on certain kilometer sections. This fixation on the measurement data can lead to an excessive focus on the technical aspects of training and affect the actual motivation to train, individual performance and health as well as the perception of one's own body feeling. In line with this, one athlete reports the following from his everyday training: “Exactly, another thing is to reach certain marks in training that are not actually important. For example, a long bike ride, then completing the 100 kilometers or, I don't know, running, completing a half marathon or things like that, which don't really make sense in terms of training, but which you only do for the numbers”. (T4, lines 428–433). If there are also inaccuracies in the measurement, e.g., due to unstable GPS signals or if targeted goals are not achieved or confirmed digitally, this can lead to dissatisfaction, pressure and technostress. One athlete backs this up with the following experience: “I ran the Berlin Marathon and pressed start and everything worked fine while I was running. [..] And you get to the finish, press stop and there's something on the clock about 41.9 kilometers. That's what really happened. And then you're standing there - I mean, you've still completed the marathon, and the joy is still great and everything is fine and dandy. But it's still a bit of a downer. “Shit, now the 300 meters”. Unfortunately, your watch doesn't praise you for running the marathon. The joy prevails, back and forth. You still ran the marathon, but somewhere along the way you lost 300 meters. I really thought for a minute about just running those 300 meters”. (T7, lines 748–759).

#### 4.2.6 Comparative pressure (codes assigned: 100)

The stress factor of comparative pressure describes the perceived urgency to compete with other athletes and surpass their performance. This dynamic goes beyond simple comparisons and includes the desire to achieve and maintain personal performance standards. This motivation can lead to a complex of negative affects, including envy, resentment and jealousy, especially in individuals with low levels of self-confidence. One athlete expresses this, for example, when she reports on her digitally determined training form and compares it to her training colleague: “so or- [Judy], she always has “top form” and I don't know when the last time I had “top form” and I always give 110 percent. When the hell was the last time I was at my best? I don't know! Maybe once?” (T1, lines 1,193–1,195). In addition, it is not only comparisons with others that play a role, but also with one's own past performances. In this context, athletes tend to present particularly outstanding training units that emphasize their own performance and convey a positive impression, while less impressive or regenerative units are often neglected. “Recording feels great, for example, when you have a long day ahead of you, training, and then you just know that when you upload it, others will see what a great training day you had today. I'm really looking forward to uploading it”. (T21 lines 309–312). The use of digital social platforms such as Strava and Garmin in particular considerably expands the area of comparability and raises internal training group comparisons to a new level, for example. One athlete, for example, reports on her early days in triathlon, when she initially spent a lot of money on training equipment and signed up for Strava as part of her newfound training motivation: “So at the beginning you stock up on your technology to keep up. Then you realize: “Wow, you're broke because you bought every-thing”. Then you realize you're not only broke, you're also bad. You realize your own status, that you can't keep up”. (T19, lines 1,232–1,235). In addition, the collection of data creates a comprehensive sporting history that shifts the focus from selective competition to permanent digital competition. One athlete, for example, describes the collection and presentation of performance data as follows: “[..] it's a ranking. Like a competition, but not a one-off competition, it's an annual ranking. An annual competition”. (T19 lines 677–679). The pressure to compare can therefore occur in a temporal, performance-related, social and digital context and, as a result, can lead to technostress.

#### 4.2.7 Permanent monitoring (codes assigned: 21)

The continuous wearing of wearables can lead to athletes being under the pressure of constant monitoring. This stress factor is accompanied by a feeling of constant evaluation and monitoring and can be intensified in particular when athletes are less able (e.g., due to injury) or less willing (e.g., due to motivational reasons) to perform. Many athletes only notice this pressure when they take off the gadget and then often report a feeling of liberation. One female athlete underpins this feeling very vividly: “And if I take the [watch] off because I have to, then I just like to use it until I put it back on, because it's just a bit more freedom. It's an active decision, because I also get messages on it sometimes. And nothing vibrates on my arm. You don't have that heaviness. It kind of pulls your wrist down. So, the watch is light, but somehow it pulls you down, figuratively speaking, because you're somehow so tied up”. (T1, lines 486–492). Another athlete answers the question of why he wants to take off his sports watch after his peak season: “Yes, simply to be completely free and just not have any pressure to train”. (T13, line 653).

#### 4.2.8 Perception discrepancy (assigned codes: 17)

The perception discrepancy describes the deviation of a person's subjective perception from the objective data determined by self-tracking devices. This stress factor occurs when a person's individual assessment of their performance, health or state of mind does not match the quantitative data recorded by technological aids. This discrepancy can lead to technostress, as it creates uncertainty and conflict when athletes have to question their own feelings and perceptions. For example, one athlete reported on the evaluation of her training status: “When it says “loss of form”, I feel the pressure. If it wasn't for this display, I wouldn't feel like this at all. You know, I train six times a week and have a rest day. I wouldn't feel like I was losing my form”. (T19, lines 802–805).

Stress can arise in particular when trying to understand and cope with the discrepancy between personal experiences and the information conveyed by technology. This can lead to increased cognitive and emotional strain. Another athlete describes this discrepancy as follows: “[..] then you might have a sleep rating of 65%, even though you wake up completely rested in the morning and think: “Great day!”, then you look at your watch and think: “Shit day!” You are advised to get more rest. “Your ability to concentrate is not so high”. And you think to yourself: “Come on, watch, leave me alone!” That might not feel so good”. (T6, lines 268–273).

## 5 Discussion

The results of the present study show that self-tracking can trigger technostress in athletes in very different ways. On the one hand, the results confirm categories from research on professional and private stress factors for technostress, but on the other hand they also reveal eight specific, new stress factors caused by self-tracking. The results are discussed below from two points of view: On the one hand, the application of the Transactional Stress Model according to Lazarus and Folkman (7) to the facts at hand is reflected upon and the resulting discussion points are addressed. On the other hand, implications for further research and practice on self-tracking in sport are presented. Finally, an outlook for further application of the results is given.

### 5.1 Theoretical implications for the transactional stress model

In this study, the transactional stress model by Lazarus and Folkman (7) was used to investigate the stress factors of technostress in the context of sport, especially in the area of self-tracking. The model proves to be particularly effective in the differentiated analysis of stress evaluation, in which it is individually assessed whether a self-tracking situation is perceived as harmful, challenging or threatening. With regard to this consideration, there is an ambivalence that underlies all the stress factors found for technostress. This ambivalence may manifest differently based on the athlete's experience level. For instance, novice athletes may feel more overwhelmed by the influx of data, while more experienced athletes may use these tools more strategically.

Self-tracking technologies, which are used as supportive tools to improve athletic performance and for health monitoring in terms of user motivation, can also be a source of significant technostress in their application. On the one hand, fitness trackers, for example, enable athletes to precisely measure their heart rate, speed and distance, which can contribute to effective training control and improved performance. On the other hand, the constant availability of this data and the need to continuously optimize performance can lead to a feeling of monitoring and pressure. The pressure to optimize performance aligns with the transactional model's distinction between “threat” and “challenge”. While some athletes may perceive performance tracking as a challenge that motivates them, others may see it as a threat to their well-being. This ambivalence is particularly evident when athletes experience excessive demands due to the constant confrontation with their measurement data.

The same applies to certain functions of the devices, such as analyzing sleep quality or measuring stress levels. These surveys, which are actually intended to promote health, can cause stress by drawing excessive attention to potential health problems or suboptimal performance levels. This illustrates how self-tracking devices, in their role as support tools, can also become stressors by blurring the boundaries between support and excessive demands.

This ambivalence reflects the dual nature of self-tracking technologies: they are both helpful and motivating as well as potentially stress-inducing and burdensome. Athletes therefore face the challenge of maximizing the benefits of these technologies while minimizing the negative psychological impact. This emphasizes the need for a balanced use and development of stress management strategies in the context of self-tracking in competitive sports.

Furthermore, the limitations of the model's applicability must be considered. Although the transactional stress model offers a basic framework for analyzing stress reactions, there is a need for adaptation in order to adequately capture the specific aspects of technostress in the sports context. In particular, the question of how the model can depict digital interactions and the resulting stress dynamics in the area of self-tracking remains unanswered. The limitation to the primary appraisal of stressors offers valuable insights, but the integration of the secondary appraisal, i.e., the assessment of individual coping capacities, could provide a more complete picture of the stress experience. In a further study, this secondary appraisal will be investigated, as the combination of primary and secondary appraisal according to the model promises valuable insights. The present study thus represents a first step, and therefore the first important findings towards understanding the facts described, but we are aware that this does not yet provide a comprehensive overall picture of technostress in the sports context.

Another aspect lies in the aspect of voluntariness. The majority of the stress factors of technostress discussed in the literature have been researched in a professional context. Here, technologies appear as obligatory work tools that are used to fulfill the work task. In contrast, in the field of triathlon and road cycling examined here, which focuses specifically on the performance-oriented leisure sector, self-measurement technologies are used on the basis of voluntary decisions. As a result of the stress-inducing stress factors identified in this study, the question arises as to what extent the voluntary decision to use self-tracking technologies influences the perception and evaluation of these stress factors. Although the stress factors confirmed so far are primarily based on mandatory use in a professional context, it is clear that similar stressors can also occur in freely chosen contexts such as competitive sport. This underlines the need to take a closer look at the specific contexts and motivations of users, as they can make a significant contribution to how technostress is experienced and processed. Voluntariness could therefore play a dual role: On the one hand, it could potentially reduce exposure to technostress as users have greater control over technology use and can customize it to their individual needs. On the other hand, it could also lead to more intensive use, especially if this is motivated by social or personal performance goals, which in turn could increase stress. Further consideration of this issue would prove useful here, which could possibly lead to an extension of the transactional stress model.

### 5.2 Learnings for self-tracking in the context of sport and further investigations

The underlying question reveals first and foremost - and this may seem trivial at first, but it is of crucial importance - that the use of self-tracking devices can cause technostress. The athletes studied perceive the stressors that trigger technostress both consciously and subconsciously. Since people strive to maintain an inner balance or restore it after a disruption, minimizing stress is crucial (7). Further studies are planned in which we want to show that athletes adapt both their behavioral and cognitive processes to reduce the stress factors caused by self-tracking.

The strength of the qualitative research design of this study lies in the contextual embedding of the information. In particular, the data collected at a training camp enabled us not only to reveal isolated stress factors, but also to shed light on their interconnectedness with everyday life and the specific demands of competitive sport. This enabled us to gain a more comprehensive understanding of technostress that goes beyond the primary appraisal level.

In a further project, we show, among other things, that performance-oriented athletes develop both emotion-oriented and problem-oriented coping strategies to reduce technostress. This manifests itself, for example, in specific behavioral patterns: 11 of the 13 athletes surveyed stated that they deliberately move less while their sports watch is charging - i.e., when no activities are being tracked - so as not to miss the recording of the movement. They find the lack of data, such as steps taken, active calories or kilometers cycled to the supermarket, stressful. The time when the watch cannot be used is therefore perceived as particularly stressful. This finding illustrates how strongly the perception of training and performance is shaped by continuous data collection. The non-existence of recordings is almost equated with the non-existence of performance, even when physical exertion takes place. This inversion of performance evaluation, in which “unseen” effort is given less value, reflects the deep rootedness of datafication in athletes’ self-perception and performance standards.

We can also show that athletes specifically adapt or modify their behavior in order to generate more advantageous results and statistics. For example, some athletes deliberately pause the recording during the run-in and run-out of a running session, or when waiting at a traffic light on a road bike, or record these phases as separate units in the recording. They do this to prevent the slower pace of these phases compared to the main part of the session from negatively affecting the overall average pace. This targeted intervention in the data recording shows how self-tracking technologies not only shape training habits, but also how deeply self-perception and performance evaluation are influenced. By actively intervening in data production, athletes consciously manipulate their performance representation, which indicates a strong internalization of performance-oriented data standards. This can further lead to a distortion of their own performance perception, where the data obscures or falsifies the athletes’ real abilities and progress.

In this context, it is particularly evident that athletes who share their activities on social platforms such as Strava think intensively about the presentation of their digital self. This manifests itself in different ways. One aspect, for example, is that athletes already reflect on how they should name the unit during the sporting activity. Particularly high-performing units are often highlighted and shared, while less impressive performances are often accompanied by justifying comments (“rode today with a headwind and hang-over”) or even removed from the public profile altogether in order to maintain a certain image. These compensation practices reflect the stress factors of “digital visibility” and “pressure to compare”, which were identified as significant sources of technostress in the results. Athletes adapt their self-presentation to meet the expectations of their digital audience, which emphasizes the importance of social recognition and external validation of their performance. This can lead to increased stress levels as athletes feel compelled to constantly document and share optimal performances, potentially undermining the authenticity of their athletic experience.

### 5.3 Applicability of the results

The results of the investigation of the stress factors of technostress through self-tracking in sport open up a variety of possible applications, ranging from training to policy design. Although the following areas of application of the identified stress factors appear intuitively relevant, it must be emphasized that these potential correlations and their effects on athletes have not yet been empirically validated. Therefore, the following explanations are to be understood as hypotheses based on the identified stress factors and could serve as a basis for future scientific studies.

The stressors of technostress in self-tracking identified in this study may be important for athletes in several aspects of their sporting activity and personal well-being. It can be hypothesized that a better understanding of these factors could help athletes to develop self-regulatory strategies. In particular, these could help to adapt the use of self-tracking devices so that they have less of a stress-inducing effect and instead improve the quality of training. It is conceivable that athletes who develop an awareness of the causes of technostress will be able to find a balance between technology-based feedback and their own physical intuition, which could potentially lead to more efficient and satisfying training.

Furthermore, awareness of specific stress factors, such as perceptual discrepancy and comparative pressure, could influence the mental health of athletes. By adapting stress management strategies, it is conceivable that athletes could potentially improve their general well-being and develop a healthier relationship with their sporting practice and the technological equipment they use. Furthermore, knowledge of these stress factors could help athletes to set more realistic goals and use self-tracking technologies in a way that positively supports their motivation and enjoyment of sport. However, this requires further research in order to derive specific instructions for action.

Coaches and sports scientists could use the insights gained to design training approaches that take into account potentially stress-inducing aspects of self-tracking technologies and thus promote a healthier training environment. In particular, methods that contribute to strengthening athletes’ body awareness and subjective perception could be crucial to develop a balanced relationship between technical feedback and physical intuition. By integrating these elements into training methods, the aim should be for athletes to learn to use and interpret technological data in a meaningful way without losing touch with their own physical sensations.

Another area of application of the present results extends to technology development. Developers of self-tracking technologies may be challenged to design systems that are more user-friendly and take into account individual needs and stress responses in order to maximize the positive aspects of self-tracking and minimize stress-inducing factors. For example, monitoring intensity, which has been identified as a stressor, could be mitigated through customizable privacy settings that allow users to set their own data management preferences. Similarly, information overload, another stress factor, could be mitigated through intelligent filtering and summary functions that allow users to receive only the information that is relevant to them. The development of personalized feedback systems could also have stress-reducing effects by taking users’ individual reactions to various data and performance indicators into account in the way data is output. If features such as these were implemented, technologies could be created that enable a customized and less stressful user experience.

The results can also be applied to healthcare and healthcare management, as health insurance companies in particular are increasingly cooperating with fitness apps to promote healthy behavior. This collaboration should be designed taking into account the stress risks that may be associated with the use of these technologies to ensure that the promotion of health does not unintentionally lead to the opposite effect.

In the policy context, the findings on stressors of technostress from self-tracking in sport could help shape policies and laws that promote healthy use of self-tracking technologies. Policy makers could be encouraged to adopt measures that not only ensure data protection and user privacy, but also take into account the psychological impact of these technologies. For example, information and data security, which has been identified as a stress factor, could be addressed through more transparent privacy policies and stronger regulation of platform companies to increase user trust in these technologies.

In addition, norms and standards for the integration of self-tracking technologies in sports and health contexts could also be established as part of the policy design to help prevent technostress. This could be done by promoting research initiatives and educational programs aimed at increasing awareness and understanding of the stress-reducing use and potential risks of self-tracking technologies. Similarly, the political debate around the use of fitness tracking in cooperation with health insurance companies could be used to strengthen health-promoting practices that go beyond mere data collection and place people at the center of technology use.

Finally, the results presented are relevant for various scientific disciplines, which allow further questions to be asked. In the social science context, the identified stress factors of technostress through self-tracking in sports provide a sound basis for investigating the effects of modern technologies on social behavior and psychological aspects in sports. Researchers could focus on questions that explore how continuous self-monitoring and social comparison processes influence athletes’ self-concept, identity and social interactions. The extent to which self-tracking technologies contribute to a shift in social norms and expectations in sport and how these shifts shape athletes’ behavior and attitudes towards training and competition could be investigated.

In sports science and training theory, stress factors are particularly relevant for the development and refinement of training approaches. Here, the focus could be on how self-tracking influences training design and management, the role of technology in performance measurement and development, and how a balanced use of self-tracking technologies can promote athletes’ physical and psychological well-being. Sports scientists could explore which specific training methods best help to reduce technostress while maximizing the benefits of data-driven training.

Interdisciplinary approaches that integrate both social science and sport science perspectives could provide more comprehensive insights into the dynamics between technostress and sport performance. This integrative research could help to develop holistic models that take into account not only the technological and physical, but also the social and psychological aspects of sport. Thus, the results would be relevant for various scientific disciplines by providing a broader understanding of the interactions between technology use and human experience in the sports context and help to provide guidelines for the healthy integration of self-tracking technologies into athletes’ training and every-day life.

## 6 Conclusion & outlook

This study focused on analyzing the stress factors of technostress that can arise from self-tracking in performance-oriented recreational sports. A total of 16 stress factors were identified. Eight of these - information overload, distraction, unavailability, loss of control, lack of sense of achievement, unreliability, complexity and self-monitoring - are known from research on technostress in the professional context and could be transferred to the sports context, adapted accordingly and thus confirmed. The study also contributed to the expansion of knowledge by identifying eight new stress factors that occur in the context of performance-oriented recreational sport: Performance enhancement imperative, lack of context, digital visibility, feedback incorporation, measurement data fixation, pressure to compare, constant monitoring and perceptual discrepancy.

A qualitative research design with an exploratory approach was used to survey the stress factors. The data was collected with the help of guided, problem-centered interviews, which allowed for an open discussion. The sample consisted of performance-oriented amateur triathletes who actively and passively engage in self-tracking. The categories of age, gender, home club and training volume or competition distance were selected in a contrastive manner. Field access was gained through the club structures of the home club, the referral of other triathletes by other club presidents and through participation in a training camp for performance-oriented long-distance triathletes. The selected research design has various limitations, including the distortion of response behavior due to social desirability and interviewer effects. In addition, the survey using an interview guide leads to a lower comparability of the results.

The stress factors of technostress identified in this study provide a basis for future research projects. The analysis was limited to the primary appraisal of potential stressors within the transactional stress model, which is why all subsequent levels of the model, such as coping processes, were not integrated into the study. Therefore, investigating the coping strategies that athletes use to develop problem- and emotion-oriented approaches to reduce technostress is a key area for further study. This future research could provide crucial insights into the effectiveness of different coping strategies in the context of performance-oriented sport. In addition, the results provide valuable clues for practical application to optimize training programs and self-tracking methods in competitive sports. The findings can help to raise awareness of the triggers of technostress in order to develop targeted stress reduction measures that are tailored to both individual and team-oriented needs.

## Data Availability

The raw data supporting the conclusions of this article will be made available by the authors, without undue reservation.
